# Arrhythmogenic Left Ventricular Cardiomyopathy: From Diagnosis to Risk Management

**DOI:** 10.3390/jcm13071835

**Published:** 2024-03-22

**Authors:** Alfredo Mauriello, Anna Selvaggia Roma, Antonia Ascrizzi, Riccardo Molinari, Francesco S. Loffredo, Antonello D’Andrea, Vincenzo Russo

**Affiliations:** 1 Cardiology Unit, Department of Medical Translational Science, University of Campania “Luigi Vanvitelli”—“V. Monaldi” Hospital, 80126 Naples, Italy; annasroma08@gmail.com (A.S.R.); antonia.ascrizzi@gmail.com (A.A.); riccmolinari@gmail.com (R.M.); francesco.loffredo@unicampania.it (F.S.L.); antonellodandrea@libero.it (A.D.); vincenzo.russo@unicampania.it (V.R.); 2 Unit of Cardiology, “Umberto I” Hospital, 84014 Nocera Inferiore, Italy

**Keywords:** sudden cardiac death, cardiac imaging, risk stratification, arrythmia, cardiomyopathy

## Abstract

Purpose of Review: Left ventricular arrhythmogenic cardiomyopathy (ALVC) is a rare and poorly characterized cardiomyopathy that has recently been reclassified in the group of non-dilated left ventricular cardiomyopathies. This review aims to summarize the background, diagnosis, and sudden cardiac death risk in patients presenting this cardiomyopathy. Recent Findings: Although there is currently a lack of data on this condition, arrhythmogenic left ventricular dysplasia can be considered a specific disease of the left ventricle (LV). We have collected the latest evidence about the management and the risks associated with this cardiomyopathy. Summary: Left ventricular arrhythmogenic cardiomyopathy is still poorly characterized. ALVC is characterized by fibrofatty replacement in the left ventricular myocardium, with variable phenotypic expression. Diagnosis is based on a multiparametric approach, including cardiac magnetic resonance (CMR) and genetic testing, and is important for sudden cardiac death (SCD) risk stratification and management. Recent guidelines have improved the management of left ventricular arrhythmogenic cardiomyopathy. Further studies are necessary to improve knowledge of this cardiomyopathy.

## 1. Introduction

Arrhythmogenic cardiomyopathy (ACM) is an inherited myocardial disease with a prevalence of 1–5000 in the general population. ACM is often associated with a mutation in genes encoding desmosome proteins, which are involved in the mechanical coupling of cardiomyocytes. ACM is characterized by the presence of both structural (fibrofatty or fibrous infiltration) and functional alterations, which are responsible for electric instability leading to ventricular arrhythmias and sudden cardiac death (SCD) [1,2].

ACM has been initially considered as a right heart cardiomyopathy because only forms with exclusive involvement of the right ventricle (RV) had been described [3,4]. Further studies, including the role of genotype–phenotype interaction and improved tissue characterization with cardiac magnetic resonance (CMR), showed the involvement of the left ventricle (LV), alone or together with the RV. The forms with LV involvement have only been classified as arrhythmogenic left ventricle cardiomyopathy (ALVC) [5,6,7].

The latest European Society of Cardiology (ESC) guidelines on cardiomyopathies recommend an approach based on the predominant phenotype at diagnosis, thus including ALVC in the group of non-dilated left ventricular cardiomyopathies (NDLVC) [8]. 

ALVC is primarily due to pathogenic variants in non-desmosomal genes, different from ACM, as those are involved in dilated cardiomyopathy (DCM) [9,10,11]; therefore, ALVC and DCM may represent the opposite ends of a broad spectrum of diseases [12,13,14]. ALVC is primarily characterized by fibrotic replacement, resulting in a normal or mildly dilated left ventricle (LV). This anatomical–pathological substrate increases the incidence of ventricular arrhythmias [7] and the risk of SCD, which may be the first clinical manifestation of the disease [15].

Several genetic and non-genetic diseases share significant clinical and imaging features with ALVC, such as DCM, myocarditis, or cardiac sarcoidosis [16,17].

Our review aims to describe the latest developments in diagnosing ALVC and the current management of ALVC patients at increased risk of developing life-threatening ventricular arrhythmias and SCD.

## 2. Diagnosis

The diagnosis of ACM is based on a multi-parametric approach according to the Padua criteria, which include: functional and morphological abnormalities investigated by echocardiography, cardiac magnetic resonance late gadolinium enhancement (CMR LGE) technique or angiography, tissue characterization findings by the CMR LGE technique, electrocardiographic depolarization and repolarization abnormalities, ventricular arrhythmias, and family or genetic background [15,18].

A “definite” diagnosis requires the fulfilment of two major or one major and two minor or four minor LV diagnostic criteria from different categories [15]. A non-definite diagnosis (“borderline” or “possible”) is given with a lower total number of diagnostic criteria fulfilled [15]. Patients with a non-definite diagnosis should be followed for disease progression over time to potentially meet the criteria for a definite diagnosis [15].

The diagnosis of ALVC is established in the presence of LV structural or functional abnormalities, by echocardiography or CMR, and a pathogenic or likely pathogenic mutation of the ACM-causing gene, without right ventricular involvement [15]. The diagnosis of the “biventricular” variant requires the presence of ≥1 morpho-functional and/or structural abnormalities in both the RV and the LV because of the lack of a disease-specific phenotype [15]. 

### 2.1. Echocardiography

Echocardiography is the primary imaging modality and the most frequently employed imaging tool in the diagnosis and follow-up of patients with ACM [16]. ALVC is characterized by a progressive worsening of the ventricular function due to the involvement of multiple LV segments. The distribution and speed of these structural abnormalities are influenced by both genetic mutations and disease stage.

**Figure 1:**
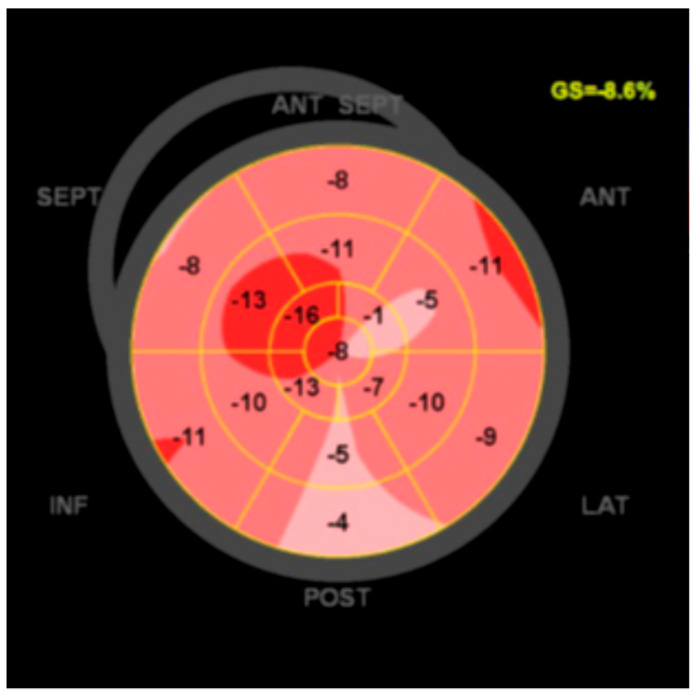
Bull’s eye plot with reduced GLS in an ALVC patient. Red ares are functionally better than lighter areas.

The LV systolic dysfunction, defined as decreasing in global LV ejection fraction (<54%) or global LV longitudinal strain (GLS) (<−18%) with or without LV dilatation [15], is considered a minor morpho-functional diagnostic criterion because of its low disease-specificity (Figure 1). 

Segura-Rodriguez et al. evaluated the layer-specific GLS to identify ACM patients at high risk of SCD. Among 45 ACM patients, those with nsVT and LGE had lower GLS values in the epicardial and mesocardial layers compared to others. Specific epicardial GLS cut-off values of −15.4% and −16.1% may identify ACM patients with nsTV and LGE, respectively [19]. Including epicardial GLS in routine echocardiographic assessments may improve the sensitivity in identifying individuals at risk of ventricular arrhythmias and, consequently, SCD.

### 2.2. Cardiac Magnetic Resonance

CMR is indicated when ALVC is suspected at initial evaluation, in order to identify the fibrofatty myocardial replacement through LGE tissue characterization [16,20,21,22]. Cine sequences are utilized for the assessment of ventricular volumes and function. T1-weighted images are used to detect fatty infiltration and LGE sequences for identifying myocardial fibrosis. 

In ALVC patients, the LV fibrotic replacement progresses from subepicardial to subendocardial layers, resulting in transmural fibrotic lesions and extensive wall thinning [23]. The CMR-detected fibro-fatty infiltration is typically observed in the basal inferolateral and anterolateral walls, followed by the mid-inferoseptal, inferolateral, and anterolateral walls [24]. 

A ring-like pattern of LGE ≥ 3 segments confirmed in two orthogonal views is considered a major criterion for ALVC and it is usually linked to genetic defects in desmoplakin (DSP), filamin C (FLNC), and phospholamban (PLN) [17,25,26,27]; segmental LGE affecting up to two segments is a minor criterion.

The LGE pattern at RV insertion points, known as the ‘septal junctional’ pattern, although observed, is not diagnostically significant for ALVC [9,16,26,27,28,29,30]. The presence of fatty tissue, detectable by CMR or multi-detector computed tomography (MDCT), in conjunction with LGE, can enhance diagnostic specificity [31].

CMR sequences like native T1 mapping and extracellular volume (ECV) quantification are capable of identifying augmented interstitial space in individuals diagnosed with ALVC [32]. 

The T2 CRM sequences are not typically recommended for diagnosing ALVC; however, they can be helpful in identifying ACM patients who exhibit symptoms resembling myocarditis, such as chest pain and elevated troponin. These symptoms are frequently present in individuals with DSP gene mutation [33,34]. 

CMR can be useful to assess the arrhythmic risk and to predict adverse outcomes in patients with ALVC [35]. A diffuse LGE at CRM is correlated with a high risk of life-threatening ventricular arrhythmias [36] and it is considered a marker of SCD risk in patients with left ventricle ejection fraction (LVEF) between 35% and 50% [11,37,38,39,40]. Autoptic studies confirmed signs of inflammation in 60–80% of ACM cases [41].

In patients with advanced ALVC additional CMR parameters such as LV myocardial deformation and dyssynchrony using CMR-feature tracking (CMR-FT) have been demonstrated to be independent risk factors for cardiovascular and arrhythmic events [39]. 

**Figure 2:**
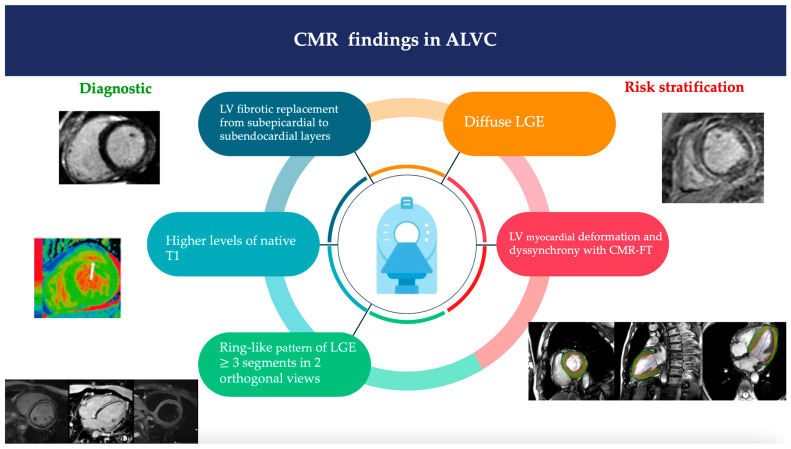
CMR findings in ALVC. Blue, light blue and green are used for diagnostic criteria; orange and red are used for risk stratification.

Figure 2 shows the most common CRM findings in AVLC patients.

### 2.3. Other Imaging

Positron emission tomography with 18F-fluorodeoxyglucose (FDG-PET) is an imaging technique used to assess myocardial inflammation. 

In an observational study by Tessier et al., which included 17 AVLC patients, 58% of the study population showed abnormal myocardial FDG uptake with varying patterns (diffuse, focal, or patchy). These findings suggest a possible link between myocardial inflammation and genetic ALVC; in particular, the specific variants in LMNA-encoded lamin A/C were more prevalent in those with abnormal FDG-PET results [42]. 

Additionally, a group of ALVC patients with FDG-PET uptake have also reported sVT, providing further evidence for the involvement of myocardial inflammation in the development of the disease [43].

### 2.4. Twelve-Lead Electrocardiography

The presence of isolated negative T waves in V4–V6, with or without the involvement of inferior leads and in the absence of left bundle branch block (LBBB), is considered a minor criterion for ALVC [17,42,44,45,46]. However, it should be noted that its specificity is limited, particularly in individuals of Afro-Caribbean ethnicity and among athletes [44,45,46]. A major criterion for ALVC is the pattern of low QRS voltages in limb leads (<0.5 mV in all limb leads), indicating replacement of the sub-epicardial LV myocardium by scar tissue [17]. To ensure accuracy, it is important to exclude other potential causes such as cardiac amyloidosis, emphysema, pericardial effusion, or obesity [17,23].

A European consensus emphasizes the use of settings for low band-pass filters (<100 Hz) to avoid false QRS voltage attenuation [4,5,15,23,26,27,30,47,48,49,50].

**Figure 3:**
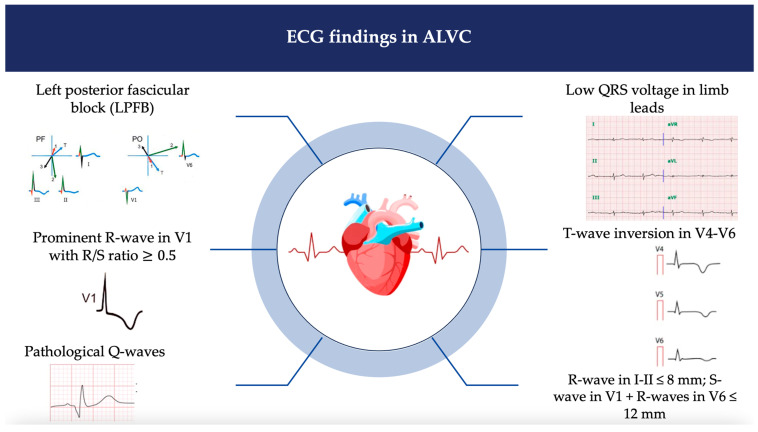
ECG findings in ALVC. PF: frontal plane; PO: horizontal plane. Symbols are the vectors of myocardial activation. Numbers are the activation order of the vectors. Different colors are used to differentiate the vectors. I, II, III, aVR, aVL, aVF, V4, V5, V6 are derivations of ECG.

The left posterior fascicular block (LPFB) has been proposed as a potential ECG marker of ALVC [51]. It seems to be more frequent in young patients with sudden cardiac death or aborted cardiac arrest compared to healthy individuals; moreover, patients with LPFB who underwent CMR showed late gadolinium enhancement in the LV, indicating fibrous/fibro-adipose myocardial replacement [47]. Other ECG signs such as the sum of R-wave amplitudes in DI and DII ≤ 8 mm and the sum of S-wave in V1 plus R-waves in V6 ≤ 12 mm are considered highly indicative of ALVC [47]. Figure 3 summarizes the main ECG findings in ALVC.

### 2.5. Holter ECG and Signal-Avereraged ECG

The European guidelines emphasize the need for a comprehensive evaluation of ventricular arrhythmias in diagnosing ALVC [17]. The absolute number and the complexity of premature ventricular beats (PVBs), as well as the PVBs’ morphology should be assessed at exercise testing or twelve-lead 24-h Holter monitoring [17]. 

**Figure 4:**
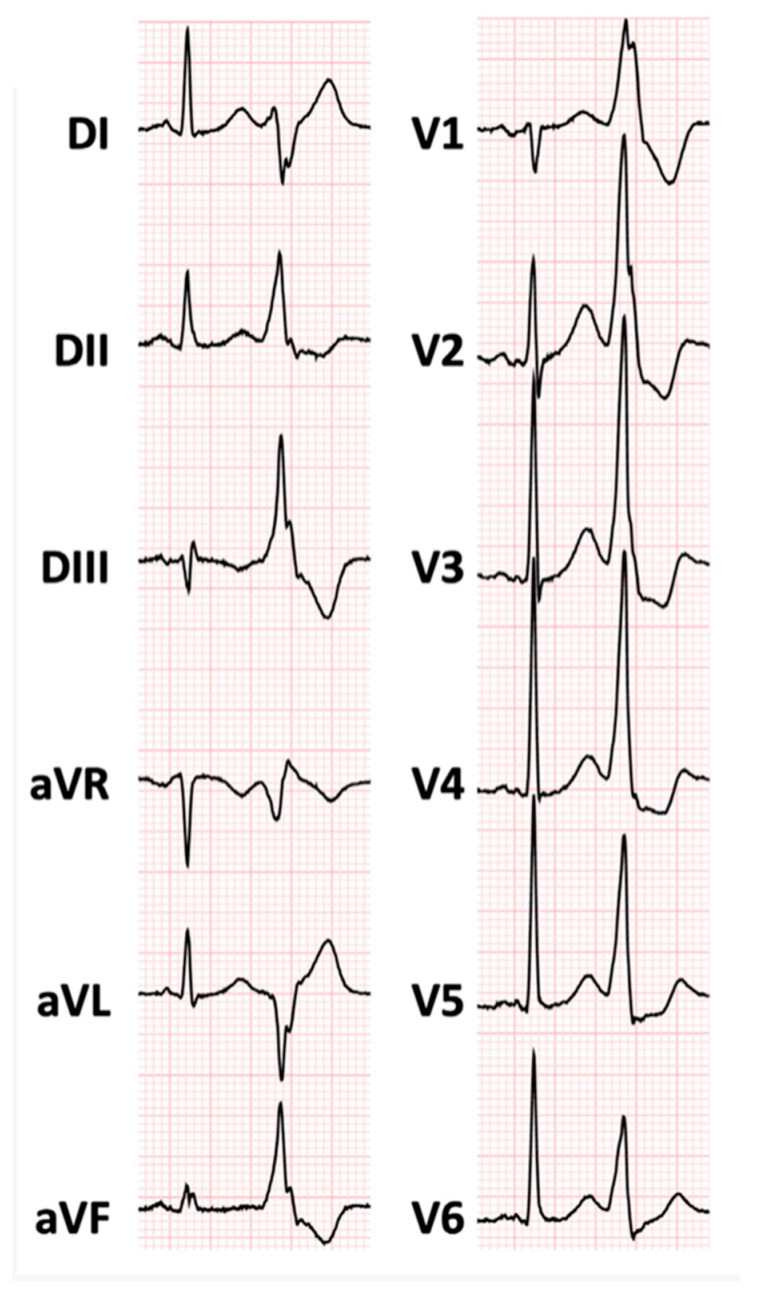
Premature ventricular contraction with right bundle branch block morphology. DI, DII, DIII, aVR, aVL, aVF, V1, V2, V3, V4, V5, V6 are derivations of ECG.

PVBs or VT with a right bundle branch block (RBBB) morphology may indicate an origin from the LV (Figure 4) [52]. More specifically, an RBBB pattern with a superior axis, a broad QRS positive in V1, and a late precordial transition to negative QRS (beyond V3) is often associated with an LV scar involving the lateral or inferolateral wall [26,53,54]. However, it is worth noting that the RBBB morphology induced by exercise is not highly specific to the underlying disease or chamber of origin.

The presence of more than 500 PVCs or ventricular arrhythmias with RBBB morphology at rest or during exercise is classified as a minor diagnostic criterion due to the low specificity [17]. Exercise-related ventricular arrhythmias can also be induced by other conditions, such as catecholaminergic polymorphic ventricular tachycardia or ischemic heart disease [17,55]. It should be noted that idiopathic ‘fascicular’ arrhythmias, which are characterized by a typical morphology of RBBB and narrow QRS, are not considered a criterion for the diagnosis of ALVC. Late potentials on signal-averaged electrocardiogram (SAECG) are no longer considered a standard diagnostic tool due to their diminished diagnostic accuracy when compared to contemporary tests [16,55]. However, SAECG may have a potential role in risk stratification for ACM and could be used to identify potentially arrhythmogenic ventricular scars [17]. Further studies are necessary to correlate the SAECG measurements and the electroanatomic mapping in order to define the arrhythmogenic substrates, in particular in patients with clinically documented sustained ventricular tachycardia [56,57,58].

### 2.6. Genetic Findings and Family History

The role of genetic testing in the diagnosis of ACM is increasing. Expert recommendations suggest that genotyping should be used to detect pathogenic or likely pathogenic mutations in probands with consistent phenotypic features of ALVC, followed by cascade screening in family members.

The 2015 American College of Medical Genetics and Genomics (ACMG) classification is used to categorize pathogenic (major criterion) and likely-pathogenic variants (minor criterion) [59]. The criteria for family history are met through pathology confirmation or diagnostic criteria in the first-degree (relative major criterion) or second-degree relative (minor criterion). Premature SCD (<35 years old) in a first-degree relative due to suspected ACM is considered a minor criterion [16,17,60].

**Figure 5:**
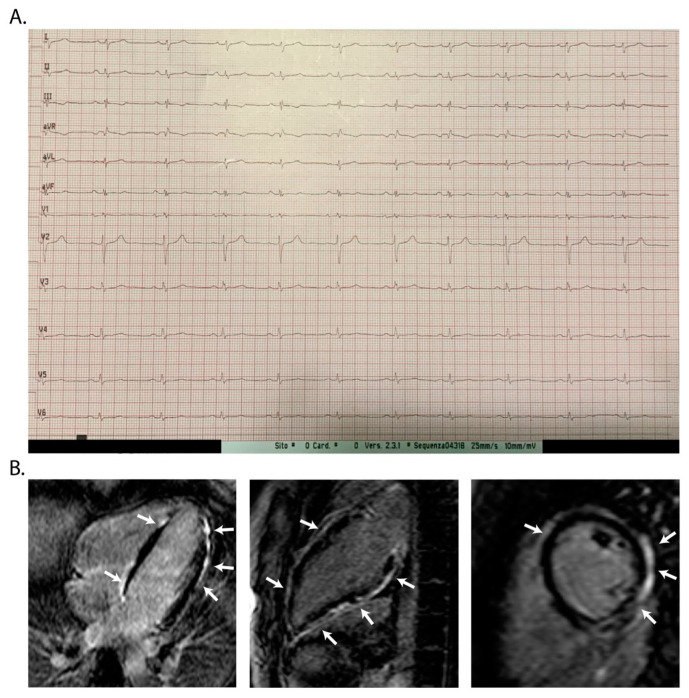
Representative ECG and CMR findings in a patient with a pathogenic DSP gene variant. (**A**) ECG showing low QRS voltages on surface 12-lead ECG together with QRS fragmentation, often present in advanced stages of ALVC when the fibrotic replacement has greatly reduced the amount of myocardial tissue of the LV. (**B**) Post-contrast CMR images in long-axis 4-chamber and 2-chamber view showing diffuse LV subepicardial LGE (white arrows); in the short-axis view, a typical ring-like distribution of LGE involving the free wall and septum of LV (white arrows). ECG findings in ALVC. DI, DII, DIII, aVR, aVL, aVF, V1, V2, V3, V4, V5, V6 are derivations of ECG.

Most of the pathogenic mutations in ALVC involve genes encoding structural proteins related to the organization of intercellular junctions [5,27,29,61]. These genes primarily affect cardiac desmosomal proteins, specifically plakophilin-2 (PKP2) [5,62], desmoplakin (DSP) (Figure 5) [27,62], desmoglein-2 (DSG2) [5], and desmocollin-2 (DSC2) [5].

ACM-causing mutations have also been identified in genes outside the desmosome, including phospholamban (PLN) [5,63], filamin C (FLNC) [9,27,28], desmin (DES) [64], titin (TTN) [27], and lamin A/C (LMNA) [27], which are linked with other cardiomyopathies, including DCM and neuromuscular cardiomyopathies. Causal mutations in non-desmosomal genes, such as transmembrane protein 43 [5] and transforming growth factor beta-3 (TGFß-3) [5], have been infrequently detected.

Desmin is the major intermediate filament protein of all three muscle cell types and connects different cell organelles. The N-terminal part of the 1A coil domain is a hot spot for pathogenic desmin mutations, which cause a filament assembly defect. A study investigated a newly discovered mutation in the DES gene known as p.Glu401Asp [64]. This mutation has been associated with arrhythmogenic cardiomyopathy/dysplasia (ARVC/D) with primary LV involvement in a Spanish family [64]. The family members carrying this mutation experience severe clinical events such as sudden cardiac death, heart failure, and arrhythmia, without affecting the skeletal muscles or conduction system [64].

ACM associated with the laminin gene may result in both mild ventricular dysfunction and severe LV function impairment [53,65,66,67,68].

A study found that in patients with ALVC and PLN mutation, both LVEF and LV mechanical dispersion can be used to stratify the risk of SCD. Patients with LVEF < 45% had a high percentage of ventricular arrhythmia and were classified as high-risk, while those with LVEF > 45% and normal LV mechanical dispersion (LVMD < 45 ms) were classified as low-risk. Patients with preserved LVEF but LV mechanical dysfunction (LVMD > 45 ms) had an intermediate risk [69].

The most common pathogenic mutations in ALVC patients are reported in Table 1.

## 3. Differential Diagnosis

ALVC shares clinical and imaging features with several genetic and non-genetic conditions, such as DCM, myocarditis, cardiac sarcoidosis, or neuromuscular cardiomyopathy [1,43]. It is important to consider alternative, specific etiologies in patients presenting features suggestive of ALVC, although the distinction between these conditions could be challenging. Clinical presentation, family history, and multimodality imaging techniques are useful to perform an adequate differential diagnosis, keeping in mind that the diagnostic confirmation of ALVC always requires genetic testing reporting the presence of a pathogenic variant in an ACM-related gene.

### 3.1. Dilated Cardiomyopathy (Early Stage)

Different grades of LV systolic impairment and myocardial fibrosis could be found both in DCM and ALVC, although there are several differences between these two conditions [1,43].

ALVC is considered a spectrum of the NDLVC, usually presenting with normal or mildly reduced systolic function or possible regional contractile abnormalities, in the absence of or with minimum LV dilation; in contrast, the DCM is characterized by a consistent global contractility dysfunction with a dilated LV [7,70].

The NDLVC form is defined as the presence of non-ischemic LV scarring or fatty replacement regardless of the presence of global or regional wall motion abnormalities (RWMASs) or isolated global LV hypokinesia without scarring. In the past, this latter phenotype was known as hypokinetic non-dilated cardiomyopathy (HNDCM) [7,70].

HNDCM could represent an early phase of both DCM and ALVC, even if clinical features, such as the presence of VA and myocardial fibrosis distribution at CMR, differ between the two conditions.

In the AVLC, the fibrofatty replacement involving the myocardium is present at the initial stage of the disease and it is considered the main determinant of the LV degenerating process; in contrast, in the HNDCM, the myocardial fibrosis occurs in the advanced stage of the disease, due to a primary myocyte contractility impairment which induces compensatory myocardial eccentric remodeling [7,71]. This also explains the different onset times of VA, detectable from the very early stage in ALVC (of note, sudden cardiac death due to malignant arrhythmias could be the first manifestation of the disease [1]) and only in the terminal phase of DCM (mainly because of severe LV dysfunction).

The confirmation for the diagnosis of ALVC remains the identification of a disease-causing gene mutation associated with an ALVC phenotype [16].

### 3.2. Cardiac Sarcoidosis

**Figure 6:**
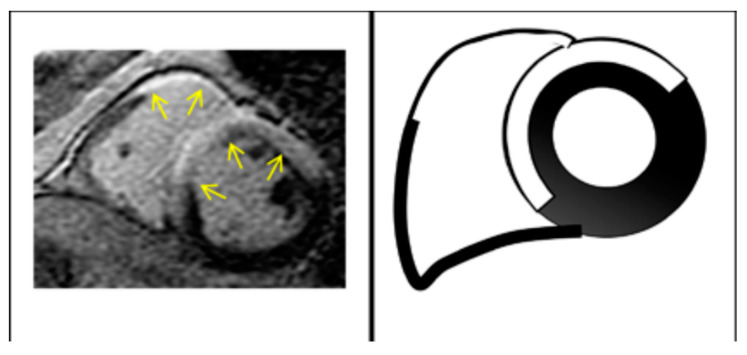
Intense LGE signals and prominent involvement of insertion points with direct and contiguous extension across the septum into RV (“hook sign”) typical of CS. Arrows indicate areas of edema.

Sarcoidosis is a systemic inflammatory disorder of unknown etiology with cardiac involvement, in particular LV, resulting in contractile dysfunction, heart failure, and life-threatening arrhythmias [72,73]. Cardiac sarcoidosis (CS) shares some of its major and minor diagnostic criteria with ALVC (i.e., LV dysfunction or VA), making the differential diagnosis difficult. The CMR is of pivotal importance since the regional distribution of fibrosis differs from ALVC; patients with CS show at CRM the “hook sign” pattern, characterized by late gadolinium enhancement in the septum continuing to ventricular insertion points and right ventricular free wall [74] (Figure 6).

At 18-FDG PET, CS patients show an uptake pattern overlapping the LGE distribution at CMR which confirms the presence of active inflammation, suggestive of CS [74].

Regarding the electrocardiographic findings, CS is more commonly associated with atrioventricular conduction disorders, even if VA may be present (Table 2).

The gold standard method for the diagnosis of CS is the endomyocardial biopsy which shows non-caseating epithelioid granulomas. In about one-quarter of cases, CS is associated with extra-cardiac manifestations, such as lung fibrosis or involvement of peripheral lymph nodes (particular attention to mediastinal lymphadenopathy), skin, and liver, all features that need to be investigated [74].

### 3.3. Chronic Myocarditis

Myocarditis is an inflammatory disease of the myocardium with various etiologies (infective, exposure to toxic substance, autoimmune disease) that shows a wide spectrum of clinical presentations, from asymptomatic silent inflammation to non-dilated cardiomyopathy, with or without HF symptoms, or life-threatening arrhythmias/SCD [75].

Clinical suspicion is provided by echocardiography which often shows a dilated or non-dilated hypokinetic LV, and CMR can confirm the diagnosis with the finding of fibrosis on LGE sequences. In this case, the differential diagnosis between chronic myocarditis and other forms of left ventricular hypokinesia, such as ALVC, is difficult.

Although the execution of CMR is mandatory, it cannot distinguish with certainty between ALVC and post-myocarditis fibrosis distribution. To achieve a correct differential diagnosis, genetic testing should be performed in patients with presentation of acute myocarditis who met at least one of the following diagnostic criteria: family history for cardiomyopathies or SCD; severe clinical presentation irrespective of age; associated clinical features (echo or CMR) related to arrhythmogenic cardiomyopathy [63].

### 3.4. Neuromuscular Disorders

Neuromuscular disorders (NMDs) are a heterogeneous group of inherited disorders affecting skeletal and cardiac muscle. In several forms of NMDs, cardiac dysfunction occurs, and cardiac disease may even be the predominant manifestation of the underlying genetic myopathy. The cardiac involvement is due to progressive interstitial fibrosis and fatty replacement in both the atria and ventricles, which may lead to cardiomyopathy, conduction defects, and tachyarrhythmias. The severity and onset of cardiac complications vary significantly across classes of NMDs. Differential diagnosis should be guided by the systemic signs specific to each dystrophy and confirmed by genetic testing [76,77].

#### 3.4.1. Dystrophinopathies

Dystrophinopathies are X-linked recessive disorders caused by mutations in the dystrophin gene (Xp21), encoding for the sarcolemma protein dystrophin virtually present in all tissues, but mostly in skeletal muscle cells and cardiomyocytes. The spectrum of dystrophinopathies embraces different clinical pictures: from Duchenne muscular dystrophy (DMD, OMIM: 310200) to Becker muscular dystrophy (BMD, OMIM: 300376). DMD and BMD both arise from a mutation in the dystrophin gene but differ in that DMD is characterized by the near absence of dystrophin, whereas in BMD, dystrophin is reduced in size and/or amount.

Dystrophin deficit cardiomyopathy is characterized by a thinner LV wall and progressive LV dilatation, reflecting the ongoing myocyte loss [78]. In particular, the repetitive mechanical stress leads to apoptosis, fibrotic substitution, and scarring that proceeds from the epicardium to the endocardium, starting generally at the region behind the posterior and mitral valve apparatus. This scarring spreads downward progressively toward the apex and around the heart, ultimately leading to DCM [79].

Dilated cardiomyopathy can be the initial presentation of DMD in the first and second decades; this presentation is later, in the third and fourth decades, in BMD patients.

Supraventricular arrhythmias, including atrial fibrillation, and premature ventricular complexes are part of the dystrophinopatic cardiomyopathy.

In DMD patients, life-threatening arrhythmias, such as ventricular tachycardias, are more frequent in the end stage of cardiomyopathy [1]. Bradyarrhythmias are relatively rare in DMD/BMD patients and can be found in the advanced stage of the disease [80,81,82,83,84].

In DMD/BMD patients, echocardiography usually shows non-ischemic regional wall motion abnormalities, left ventricular dilation, and reduced global systolic function. At CRM, the LGE pattern shows a typical distribution in the subepicardial layer of the LV posterobasal region, which is consistent with the pathological findings of fibrosis in the inferobasal wall [85,86,87].

#### 3.4.2. Emery–Dreifuss Muscular Dystrophy (EDMD)

Emery–Dreifuss muscular dystrophy (EDMD) is a neuromuscular disorder caused by mutations in genes encoding nuclear envelope proteins, such as lamin-A (LMNA) (OMIM: 310300) and emerin (EMD) genes (OMIM: 181350).

EDMD is characterized by the clinical triad of joint contractures that begin in early childhood and slowly progressive muscle weakness and wasting initially in a humero-peroneal distribution that later extends to the scapular and pelvic girdle muscles. The cardiac involvement in patients with EMDM associated with laminopathy is typically characterized by atrioventricular conduction abnormalities, atrial fibrillation/flutter, atrial standstill, and life-threatening cardiac arrhythmias; heart remodeling towards dilated or restrictive cardiomyopathy appears in the late stage of disease [88]. Little is still known about cardiac involvement in EDMD patients associated with emerinopathy [89,90,91,92,93].

At CMR, patients with LMNA mutation show an LGE pattern with a typical distribution in the basal or mid-ventricular septal wall [90].

#### 3.4.3. Myotonic Dystrophy Type 1 (DM1)

DM1 (OMIM: 160900) is an autosomal dominant disorder with incomplete penetrance and variable expressivity. It is caused by the 3′untranslated region of the Dystrophia Myotonica Protein Kinase (DMPK) gene. DM1 is a multi-system disorder characterized by muscle wasting, myotonia, cardiac and pulmonary involvement, and neuropsychological dysfunction [94].

Cardiac involvement is reported in about 80% of cases and often precedes muscular impairment. The anatomy–pathologic substrate consists of progressive fibrosis and fatty replacement of the myocardium, involving both the conduction system and the atrial and ventricular myocardium. The DM1 cardiac phenotype is broad and includes conduction disturbances, arrhythmias, and subclinical diastolic and systolic dysfunction in the early stage of disease; in contrast, severe ventricular systolic dysfunction occurs in the late stage of disease. In fact, dilated cardiomyopathy and end-stage cardiomyopathy are uncommon [95,96,97,98]. SCD occurs in 30% of DM1 patients [99,100,101,102,103,104].

### 3.5. Chagas Disease

Chagas disease (CD) is an inflammatory, infectious disease caused by the parasite Trypanosoma Cruzi. After an initial asymptomatic or oligosymptomatic phase with fever, anorexia, and tachycardia, about 30% of patients may progress to the chronic phase with neurological, cardiac, and digestive disorders. Cardiac involvement is the most serious manifestation of CD and is characterized by both arrhythmias, in particular AV block and cardiomyopathy. Chronic chagasic cardiomyopathy (CCC) is characterized by diffuse myocarditis, with tissue substitution by fibrosis and segmental wall motion abnormality [105,106], with dilated cardiomyopathy with HF being considered the late stage of clinical progression [107]. The segments more often involved in this case are the posterolateral and apical walls and the grade of dysfunction can vary from an akinesia to an aneurysmatic dilation with possible thrombotic formations [108,109].

Besides the execution of CMR, a common feature of Chagas disease detectable by iodine-123 metaiodobenzylguanidine (MBG) scintigraphy is the presence of parasympathetic denervation, which is not found in ALVC [110]. In the presence of these features, to discriminate Chagas disease from other conditions causing LV dilation, epidemiological contest should be investigated and laboratory research for the etiological pathogen will give the diagnostic confirmation.

## 4. Genotype–Phenotype Correlation for SCD Prevention Strategy

SCD prevention among patients with ALVC and normal or mildly impaired LVEF is still challenging. The genotype seems to play a significant role in predicting the risk of SCD; in particular, patients carrying variations in specific genes such as PLN, TMEM43, DES, DSP, LMNA, and truncating variants of FLNC and RBM20 (OMIM: 613172) showed higher rates of major arrhythmic events irrespective of their LVEF [62,63,105,106,107,108,109]. When available, specific risk estimation tools tailored to high-risk genotypes have to be utilized [7,53,110,111,112,113,114,115,116,117,118].

The LMNA-risk VTA calculator aims to estimate the 5-year risk of sudden cardiac death, appropriate ICD therapy, or other manifestations of hemodynamically unstable VTA. The model integrates five variables, namely, sex, non-missense LMNA mutation, AVB, nsVT, and LVEF as risk factors [119]. AV conduction anomalies, nsVTs, and LVEF showed the strongest association with arrhythmic events in the model [119]. The threshold to guide the ICD implantation in SCD primary prevention is set at a risk of ≥7% at 5 years [7].

The variant-specific risk prediction model designed for patients with the PLN p.Arg14del offers a 5-year risk estimation for a composite endpoint including sVT, appropriate ICD therapy, and aborted SCD [63]. The model integrates LVEF, the amount of inferior or precordial leads with negative T waves, Low-voltage ECG, and the amount of PVC/24 h as risk factors. The amount of PVC/24-h and LVEF showed the strongest association with arrhythmic events in the model [63]. The threshold to guide the ICD implantation in SCD primary prevention is set at a risk of ≥7% at 5 years [7].

In TMEM43 mutated patients, beyond LVEF <45% and LGE at CMR, male sex, nsVT, and a burden >200 PVCs at 24 h Holter ECG are considered to be high-risk features [117,120]. In patients with a mutation in DSP, RBM20 genes, and FLNC-truncating variants, LVEF < 45% and significant LGE at CMR are correlated with arrhythmic events [115,118,121].

No data are available for [115,118,121] SCD risk stratification in ALVC patients without a causative gene variant; however, the ICD implantation in primary prevention may be considered in AVLC patients with nsVT, high burden of PVCs, syncope, family history of SCD, or significant LGE on CMR [7]. Patients should be informed about gaps in evidence and a shared decision-making process accounting for individual preferences for ICD implantation in primary prevention, evaluating the benefit/risk ratio [38,122,123,124,125,126,127,128,129,130,131,132]. Table 3 summarizes genotype-specific reccommendatios for ICD implantation.

## 5. Conclusions

ALVC is a rare condition characterized by a wide spectrum of clinical presentations, from arrhythmic to cardiomyopathy phenotype. A multiparametric approach including both imaging and genetic testing, according to the Padua criteria, is mandatory for the diagnosis. The stratification of SCD risk remains a challenge in the management of patients with ALVC.

## Figures and Tables

**Table 1:** Pathogenic mutations in ALVC.

***Desmosomal***
*PKP2—Plakophilin C (OMIM: 602861)* *DSP—Desmoplakin (OMIM: 125647)* *DSG2—Desmoglein 2 (OMIM: 125671)* *DSC2—Desmocollin (OMIM: 125645)*
***Non-desmosomal***
*PLN—Phospholamban (OMIM: 172405)* *FLNC—Filamin C (OMIM: 1029565)* *DES—Desmin (OMIM: 125660)* *TTN—Titin (OMIM: 188840)* *LMNA—Lamin A/C (OMIM: 150330)* *TMEM 43—Transmembrane protein 43 (OMIM: 612048)* *TGFB3—Transforming growth factor-3 (OMIM: 190230)*

**Table 2:** Differential diagnosis of ALVC.

	ALVC	DCM	Myocarditis	CS
LV function	- Normal or mildly reduced systolic function - Regional hypokinesia/akinesia of free wall and septum	- Systolic disfunction - Global contractility impairment	- From normal LV function to severe systolic dysfunction	- LV function impairment - May involve right ventricle
LV dilation	- Absent (HNDCM) or mildly present	- Present	- Present or absent	- Present
LGE at CMR	- Fat infiltration at basal inferolateral and anterolateral walls, or mid inferoseptal, inferolateral and anterolateral walls - Fibrosis starting from the subepicardial and ongoing to the subendocardial layer	- Absence of fibrosis at early phases - Later development of myocardial fibrosis due to compensatory eccentric remodeling	- Mid-wall non-ischemic LGE distribution	- Typical LGE of the basal septum
Peculiar features	- Subepicardial “fibrosis ring”, with LGE affecting LV free wall and septum (short axis view at CMR)	- Mid-wall non-ischemic LGE distribution involving the septum at CMR	- Absence of specific pattern	- The “hook sign” (LGE distribution at RV insertion points spreading to septum and RV) is a specific sign of CS
Tachy/Brady-arrhythmias	- Ventricular arrhythmias with RBBB morphology - Arrythmias present from the early stage	- Non-sustained or sustained ventricular arrhythmias, generally in the advanced stage	- Non sustained or sustained tachyarrhythmias	- Conduction disturbance (atrioventricular blocks and bundle branch blocks)
Further investigations	- Identification of disease-causing gene mutation associated with an ALVC phenotype at genetic testing	- Exclusion of ischemic etiology - Genetic test if “red flags” for genetic forms of DCM are present	- Genetic testing if a genetic etiology is suspected	- 18-FDG PET showing active inflammation in the same areas of LGE distribution in CMR - Endomyocardial biopsy for the research of non-caseating epithelioid granulomas

ALVC: arrhythmogenic left ventricular cardiomyopathy; CMR: cardiac magnetic resonance; CS: cardiac sarcoidosis; DCM: dilated cardiomyopathy; HNDCM: hypokinetic non-dilated cardiomyopathy; LGE: late gadolinium enhancement; LV: left ventricle; RBBB: right bundle branch block; RV: right ventricle.

**Table 3:** Genotype-specific recommendations for ICD implantation in the primary prevention of SCD in patients with ALVC (adapted from 2023 ESC task-force expert consensus on the management of cardiomyopathies [7].

Genotype	Additional Risk Factors (If Present ICD Implantation Is Recommended)	Class of Recommendation—Level of Evidence
LMNA	≥7% five-year risk of SCD estimated with LMNA-risk VTA calculator	IIa-C
FLNC-truncating variants	LGE on CMR LVEF < 45%	IIa-C
TMEM43	Male Female and any of the following: LVEF < 45%, NSVT, LGE on CMR, >200 VE on 24 h Holter ECG	IIa-C
PLN p.Arg14del variant	≥7% five-year risk of SCD estimated with PLN variant-specific risk calculator.	IIa-C
DSP	LGE on CMR LVEF < 45%	IIa-C
DES	No adjunctive risk factors	IIb-C
RBM20	LGE on CMR LVEF < 45%	IIa-C
ALVC with any known causative gene variant and LVEF > 35%	No adjunctive risk factors	IIb-C
ALVC without known causative gene variant and LVEF > 35%	Syncope LGE presence on CMR	IIb-C

ALVC: arrhythmogenic left ventricular cardiomyopathy; CMR: cardiac magnetic resonance; DSP: desmoplakin; DES: desmin; FLNC: filamin C; LVEF: left ventricular ejection fraction; ICD: implantable cardiac defibrillator; LGE: late gadolinium enhancement; LMNA: lamin A; NSVT: non-sustained ventricular arrhythmia; PLN: phospholamban; RBM20: RNA-binding motif protein 20; SCD: sudden cardiac death; TMEM43: transmembrane protein 43; VE: ventricular ectopia. Class of Recommendation II: Conditions for which there is conflicting evidence and/or a divergence of opinion about the usefulness/efficacy of a procedure or treatment. IIa. Weight of evidence/opinion is in favor of usefulness/efficacy. IIb. Usefulness/efficacy is less well established by evidence/opinion. Level of Recommendation: Level C: Consensus opinion of experts.

## Data Availability

Not applicable.

## References

[1] Elliott P.M., Anastasakis A., Asimaki A., Basso C., Bauce B., Brooke M.A., Calkins H., Corrado D., Duru F., Green K.J. (2019). Definition and treatment of arrhythmogenic cardiomyopathy: An updated expert panel report. Eur. J. Heart Fail..

[2] Corrado D., Basso C., Thiene G., McKenna W.J., Davies M.J., Fontaliran F., Nava A., Silvestri F., Blomstrom-Lundqvist C., Wlodarska E.K. (1997). Spectrum of Clinicopathologic Manifestations of Arrhythmogenic Right Ventricular Cardiomyopathy/Dysplasia: A Multicenter Study. J. Am. Coll. Cardiol..

[3] Marcus F.I., Fontaine G.H., Guiraudon G., Frank R., Laurenceau J.L., Malergue C., Grosgogeat Y. (1982). Right ventricular dysplasia: A report of 24 adult cases. Circulation.

[4] Sen-Chowdhry S., Syrris P., Ward D., Asimaki A., Sevdalis E., McKenna W.J. (2007). Clinical and Genetic Characterization of Families With Arrhythmogenic Right Ventricular Dysplasia/Cardiomyopathy Provides Novel Insights Into Patterns of Disease Expression. Circulation.

[5] Corrado D., Basso C., Judge D.P. (2017). Arrhythmogenic Cardiomyopathy. Circ. Res..

[6] Towbin J.A., McKenna W.J., Abrams D.J., Ackerman M.J., Calkins H., Darrieux F.C., Daubert J.P., de Chillou C., DePasquale E.C., Desai M.Y. (2019). 2019 HRS expert consensus statement on evaluation, risk stratification, and management of arrhythmogenic cardiomyopathy. Heart Rhythm.

[7] Arbelo E., Protonotarios A., Gimeno J.R., Arbustini E., Barriales-Villa R., Basso C., Bezzina C.R., Biagini E., Blom N.A., de Boer R.A. (2023). 2023 ESC Guidelines for the management of cardiomyopathies. Eur. Heart J..

[8] Quarta G., Syrris P., Ashworth M., Jenkins S., Alapi K.Z., Morgan J., Muir A., Pantazis A., McKenna W.J., Elliott P.M. (2012). Mutations in the Lamin A/C gene mimic arrhythmogenic right ventricular cardiomyopathy. Eur. Heart J..

[9] Ortiz-Genga M.F., Cuenca S., Ferro M.D., Zorio E., Salgado-Aranda R., Climent V., Padrón-Barthe L., Duro-Aguado I., Jiménez-Jáimez J., Hidalgo-Olivares V.M. (2016). Truncating FLNC Mutations Are Associated With High-Risk Dilated and Arrhythmogenic Cardiomyopathies. J. Am. Coll. Cardiol..

[10] Te Riele A.S.J., Agullo-Pascual E., James C.A., Leo-Macias A., Cerrone M., Zhang M., Lin X., Lin B., Rothenberg E., Sobreira N.L. (2017). Multilevel analyses of SCN5A mutations in arrhythmogenic right ventricular dysplasia/cardiomyopathy suggest non-canonical mechanisms for disease pathogenesis. Cardiovasc. Res..

[11] Zeppenfeld K., Tfelt-Hansen J., de Riva M., Winkel B.G., Behr E.R., A Blom N., Charron P., Corrado D., Dagres N., de Chillou C. (2022). 2022 ESC Guidelines for the management of patients with ventricular arrhythmias and the prevention of sudden cardiac death. Eur. Heart J..

[12] Marcus F.I., McKenna W.J., Sherrill D., Basso C., Bauce B., Bluemke D.A., Calkins H., Corrado D., Cox M.G., Daubert J.P. (2010). Diagnosis of Arrhythmogenic Right Ventricular Cardiomyopathy/Dysplasia. Circulation.

[13] Tsui H., van Kampen S.J., Han S.J., Meraviglia V., van Ham W.B., Casini S., van der Kraak P., Vink A., Yin X., Mayr M. (2023). Desmosomal protein degradation as an underlying cause of arrhythmogenic cardiomyopathy. Sci. Transl. Med..

[14] Monda E., Lioncino M., Rubino M., Caiazza M., Cirillo A., Fusco A., Pacileo R., Fimiani F., Amodio F., Borrelli N. (2022). The Risk of Sudden Unexpected Cardiac Death in Children. Heart Fail. Clin..

[15] Corrado D., Perazzolo Marra M., Zorzi A., Beffagna G., Cipriani A., Lazzari M., Migliore F., Pilichou K., Rampazzo A., Rigato I. (2020). Diagnosis of arrhythmogenic cardiomyopathy: The Padua criteria. Int. J. Cardiol..

[16] Corrado D., Van Tintelen P.J., McKenna W.J., Hauer R.N.W., Anastastakis A., Asimaki A., Basso C., Bauce B., Brunckhorst C., Bucciarelli-Ducci C. (2020). International Experts. Arrhythmogenic right ventricular cardiomyopathy: Evalu-ation of the current diagnostic criteria and differential diagnosis. Eur. Heart J..

[17] Corrado D., Anastasakis A., Basso C., Bauce B., Blomström-Lundqvist C., Bucciarelli-Ducci C., Cipriani A., De Asmundis C., Gandjbakhch E., Jiménez-Jáimez J. (2024). Proposed diagnostic criteria for arrhythmogenic cardiomyopathy: European Task Force consensus report. Int. J. Cardiol..

[18] Haugaa K.H., Haland T.F., Leren I.S., Saberniak J., Edvardsen T. (2016). Arrhythmogenic right ventricular cardiomyopathy, clinical manifestations, and diagnosis. EP Eur..

[19] Segura-Rodríguez D., Bermúdez-Jiménez F.J., González-Camacho L., Escobar E.M., García-Orta R., Alcalá-López J.E., Pavés A.B., Oyonarte-Ramírez J.M., López-Fernández S., Álvarez M. (2021). Layer-Specific Global Longitudinal Strain Predicts Arrhythmic Risk in Arrhythmogenic Cardiomyopathy. Front. Cardiovasc. Med..

[20] Te Riele A.S., Tandri H., Bluemke D.A. (2014). Arrhythmogenic right ventricular cardiomyopathy (ARVC): Cardiovascular magnetic resonance update. J. Cardiovasc. Magn. Reson..

[21] Borgquist R., Haugaa K.H., Gilljam T., Bundgaard H., Hansen J., Eschen O., Jensen H.K., Holst A.G., Edvardsen T., Svendsen J.H. (2014). The diagnostic performance of imaging methods in ARVC using the 2010 Task Force criteria. Eur. Heart J.-Cardiovasc. Imaging.

[22] Haugaa K.H., Basso C., Badano L.P., Bucciarelli-Ducci C., Cardim N., Gaemperli O., Galderisi M., Habib G., Knuuti J., Lancellotti P. (2017). Comprehensive multi-modality imaging approach in arrhythmogenic cardiomyopathy—An expert consensus document of the European Association of Cardiovascular Imaging. Eur. Heart J.-Cardiovasc. Imaging.

[23] De Lazzari M., Zorzi A., Cipriani A., Susana A., Mastella G., Rizzo A., Rigato I., Bauce B., Giorgi B., Lacognata C. (2018). Relationship Between Electrocardiographic Findings and Cardiac Magnetic Resonance Phenotypes in Arrhythmogenic Cardiomyopathy. J. Am. Heart Assoc..

[24] Bariani R., Cipriani A., Rizzo S., Celeghin R., Bueno Marinas M., Giorgi B., De Gaspari M., Rigato I., Leoni L., Zorzi A. (2021). “Hot phase” clinical presentation in arrhythmogenic cardiomyopathy. EP Eur..

[25] Russo A.D., Compagnucci P., Zorzi A., Cavarretta E., Castelletti S., Contursi M., D’Aleo A., D’Ascenzi F., Mos L., Palmieri V. (2023). Electroanatomic mapping in athletes: Why and when. An expert opinion paper from the Italian Society of Sports Cardiology. Int. J. Cardiol..

[26] Cipriani A., Bauce B., De Lazzari M., Rigato I., Bariani R., Meneghin S., Pilichou K., Motta R., Aliberti C., Thiene G. (2020). Arrhythmogenic Right Ventricular Cardiomyopathy: Characterization of Left Ventricular Phenotype and Differential Diagnosis with Dilated Cardiomyopathy. J. Am. Heart Assoc..

[27] Augusto J.B., Eiros R., Nakou E., Moura-Ferreira S., A Treibel T., Captur G., Akhtar M.M., Protonotarios A., Gossios T.D., Savvatis K. (2020). Dilated cardiomyopathy and arrhythmogenic left ventricular cardiomyopathy: A comprehensive genotype-imaging phenotype study. Eur. Heart J.-Cardiovasc. Imaging.

[28] Hall C.L., Akhtar M.M., Sabater-Molina M., Futema M., Asimaki A., Protonotarios A., Dalageorgou C., Pittman A.M., Suarez M.P., Aguilera B. (2020). Filamin C variants are associated with a distinctive clinical and immunohistochemical arrhythmogenic cardiomyopathy phenotype. Int. J. Cardiol..

[29] Segura-Rodríguez D., Bermúdez-Jiménez F.J., Carriel V., López-Fernández S., González-Molina M., Ramírez J.M.O., Fernández-Navarro L., García-Roa M.D., Cabrerizo E.M., Durand-Herrera D. (2020). Myocardial fibrosis in arrhythmogenic cardiomyopathy: A genotype–phenotype correlation study. Eur. Heart J.-Cardiovasc. Imaging.

[30] Sen-Chowdhry S., Syrris P., Prasad S.K., Hughes S.E., Merrifield R., Ward D., Pennell D.J., McKenna W.J. (2008). Left-Dominant Arrhythmogenic Cardiomyopathy: An Under-Recognized Clinical Entity. J. Am. Coll. Cardiol..

[31] Aquaro G.D., Barison A., Todiere G., Grigoratos C., Ali L.A., Di Bella G., Emdin M., Festa P. (2016). Usefulness of Combined Functional Assessment by Cardiac Magnetic Resonance and Tissue Characterization Versus Task Force Criteria for Diagnosis of Arrhythmogenic Right Ventricular Cardiomyopathy. Am. J. Cardiol..

[32] Rubino M., Scatteia A., Frisso G., Pacileo G., Caiazza M., Pascale C.E., Guarini P., Limongelli G., Dellegrottaglie S. (2021). Imaging the “Hot Phase” of a Familiar Left-Dominant Arrhythmogenic Cardiomyopathy. Genes.

[33] Bourfiss M., Prakken N.H., van der Heijden J.F., Kamel I., Zimmerman S.L., Asselbergs F.W., Leiner T., Velthuis B.K., Riele A.S.T. (2019). Diagnostic Value of Native T1 Mapping in Arrhythmogenic Right Ventricular Cardiomyopathy. JACC Cardiovasc. Imaging.

[34] He J., Xu J., Li G., Zhou D., Li S., Zhuang B., Chen X., Duan X., Li L., Fan X. (2020). Arrhythmogenic Left Ventricular Cardiomyopathy: A Clinical and CMR Study. Sci. Rep..

[35] Monda E., Frisso G., Rubino M., Caiazza M., Esposito A., Cirillo A., Fusco A., Palmiero G., Mazzaccara C., Pacileo R. (2021). Potential role of imaging markers in predicting future disease expression of arrhythmogenic cardiomyopathy. Futur. Cardiol..

[36] Tat E., Ball C., Camren G.P., Wroblewski I., Dajani K.A., Goldberg A., Kinno M., Sanagala T., Syed M.A., Wilber D.J. (2022). Impact of late gadolinium enhancement extent, location, and pattern on ventricular tachycardia and major adverse cardiac events in patients with ischemic vs. non-ischemic cardiomyopathy. Front. Cardiovasc. Med..

[37] Aquaro G.D., De Luca A., Cappelletto C., Raimondi F., Bianco F., Botto N., Lesizza P., Grigoratos C., Minati M., Dell’omodarme M. (2020). Prognostic Value of Magnetic Resonance Phenotype in Patients with Arrhythmogenic Right Ventricular Cardiomyopathy. J. Am. Coll. Cardiol..

[38] Corrado D., Wichter T., Link M.S., Hauer R.N., Marchlinski F.E., Anastasakis A., Bauce B., Basso C., Brunckhorst C., Tsatsopoulou A. (2015). Treatment of arrhythmogenic right ventricular cardiomyopathy/dysplasia: An international task force consensus statement. Eur. Heart J..

[39] Song Y., Li L., Chen X., Ji K., Lu M., Hauer R., Chen L., Zhao S. (2021). Left Ventricular Longitudinal Dyssynchrony by CMR Feature Tracking Is Related to Adverse Prognosis in Advanced Arrhythmogenic Cardiomyopathy. Front. Cardiovasc. Med..

[40] Protonotarios A., Wicks E. (2023). The role of FDG-PET imaging in arrhythmogenic cardiomyopathy. Int. J. Cardiol..

[41] Protonotarios A., Wicks E., Ashworth M., Stephenson E., Guttmann O., Savvatis K., Sekhri N., Mohiddin S.A., Syrris P., Menezes L. (2018). Prevalence of 18F-fluorodeoxyglucose positron emission tomography abnormalities in patients with arrhythmogenic right ventricular cardiomyopathy. Int. J. Cardiol..

[42] Neves R., Tseng A.S., Garmany R., Fink A.L., McLeod C.J., Cooper L.T., MacIntyre C.J., Homb A.C., Rosenbaum A.N., Bois J.P. (2023). Cardiac fludeoxyglucose-18 positron emission tomography in genotype-positive arrhythmogenic cardiomyopathy. Int. J. Cardiol..

[43] Monda E., Rubino M., Palmiero G., Verrillo F., Lioncino M., Diana G., Cirillo A., Fusco A., Dongiglio F., Caiazza M. (2023). Multimodality Imaging in Arrhythmogenic Left Ventricular Cardiomyopathy. J. Clin. Med..

[44] Calore C., Zorzi A., Sheikh N., Nese A., Facci M., Malhotra A., Zaidi A., Schiavon M., Pelliccia A., Sharma S. (2016). Electrocardiographic anterior T-wave inversion in athletes of different ethnicities: Differential diagnosis between athlete’s heart and cardiomyopathy. Eur. Heart J..

[45] D’Ascenzi F., Anselmi F., Adami P.E., Pelliccia A. (2020). Interpretation of T-wave inversion in physiological and pathological conditions: Current state and future perspectives. Clin. Cardiol..

[46] Wilson M.G., Sharma S., Carré F., Charron P., Richard P., O’Hanlon R., Prasad S.K., Heidbuchel H., Brugada J., Salah O. (2012). Significance of deep T-wave inversions in asymptomatic athletes with normal cardiovascular examinations: Practical solutions for managing the diagnostic conundrum. Br. J. Sports Med..

[47] Calò L., Oliviero G., Crescenzi C., Romeo F., Martino A., Bressi E., Stefanini M., Silvetti E., Danza L., Rebecchi M. (2023). Electrocardiogram in arrhytmogenic cardiomyopathy. Eur. Heart J. Suppl..

[48] Cadrin-Tourigny J., Bosman L.P., Nozza A., Wang W., Tadros R., Bhonsale A., Bourfiss M., Fortier A., Lie Ø.H., Saguner A.M. (2022). A new prediction model for ventricular arrhythmias in arrhythmogenic right ventricular cardiomyopathy. Eur. Heart J..

[49] Jordà P., Bosman L.P., Gasperetti A., Mazzanti A., Gourraud J.B., Davies B., Frederiksen T.C., Weidmann Z.M., Di Marco A., Roberts J.D. (2022). Arrhythmic risk prediction in arrhythmogenic right ventricular cardiomyopathy: External validation of the arrhythmogenic right ventricular cardiomyopathy risk calculator. Eur. Heart J..

[50] Platonov P.G., Calkins H., Hauer R.N., Corrado D., Svendsen J.H., Wichter T., Biernacka E.K., Saguner A.M., Riele A.S.T., Zareba W. (2016). High interobserver variability in the assessment of epsilon waves: Implications for diagnosis of arrhythmogenic right ventricular cardiomyopathy/dysplasia. Heart Rhythm..

[51] Calò L., Della Bona R., Martino A., Crescenzi C., Panattoni G., D’amati G., Gaita F., Mango R., Sciarra L., Laredo M. (2021). Left Posterior Fascicular Block and Increased Risk of Sudden Cardiac Death in Young People. J. Am. Coll. Cardiol..

[52] Laredo M., Tovia-Brodie O., Milman A., Michowitz Y., Roudijk R.W., Peretto G., Badenco N., Riele A.S.J.M.T., Sala S., Duthoit G. (2023). Electrocardiographic findings in patients with arrhythmogenic cardiomyopathy and right bundle branch block ventricular tachycardia. Europace.

[53] Corrado D., Basso C. (2022). Arrhythmogenic left ventricular cardiomyopathy. Heart.

[54] Zorzi A., Marra M.P., Rigato I., De Lazzari M., Susana A., Niero A., Pilichou K., Migliore F., Rizzo S., Giorgi B. (2016). Nonischemic Left Ventricular Scar as a Substrate of Life-Threatening Ventricular Arrhythmias and Sudden Cardiac Death in Competitive Athletes. Circ. Arrhythm. Electrophysiol..

[55] Pearman C.M., Lee D., Davies B., Khan H., Tadros R., Cadrin-Tourigny J., Roberts J.D., Sanatani S., Simpson C., Angaran P. (2023). Incremental value of the signal-averaged ECG for diagnosing arrhythmogenic cardiomyopathy. Heart Rhythm..

[56] Kumar S., Baldinger S.H., Kapur S., Romero J., Mehta N.K., Mahida S., Fujii A., Tedrow U.B., Stevenson W.G. (2018). Right ventricular scar-related ventricular tachycardia in nonischemic cardiomyopathy: Electrophysiological characteristics, mapping, and ablation of underlying heart disease. J. Cardiovasc. Electrophysiol..

[57] Bosman L.P., Cadrin-Tourigny J., Bourfiss M., Ghasabeh M.A., Sharma A., Tichnell C., Roudijk R.W., Murray B., Tandri H., Khairy P. (2020). Diagnosing arrhythmogenic right ventricular cardiomyopathy by 2010 Task Force Criteria: Clinical performance and simplified practical implementation. EP Eur..

[58] Casella M., Gasperetti A., Sicuso R., Conte E., Catto V., Sommariva E., Bergonti M., Vettor G., Rizzo S., Pompilio G. (2020). Characteristics of Patients with Arrhythmogenic Left Ventricular Cardiomyopathy. Circ. Arrhythm. Electrophysiol..

[59] Richards S., Aziz N., Bale S., Bick D., Das S., Gastier-Foster J., Grody W.W., Hegde M., Lyon E., Spector E. (2015). Standards and guidelines for the interpretation of sequence variants: A joint consensus recommendation of the American College of Medical Genetics and Genomics and the Association for Molecular Pathology. Genet. Med..

[60] Ackerman M.J., Priori S.G., Willems S., Berul C., Brugada R., Calkins H., Camm A.J., Ellinor P.T., Gollob M., Hamilton R. (2011). HRS/EHRA expert consensus statement on the state of genetic testing for the channelopathies and cardiomyopathies this document was developed as a partnership between the Heart Rhythm Society (HRS) and the European Heart Rhythm Association (EHRA). Europace.

[61] Corrado D., Link M.S., Calkins H. (2017). Arrhythmogenic Right Ventricular Cardiomyopathy. N. Engl. J. Med..

[62] Smith E.D., Lakdawala N.K., Papoutsidakis N., Aubert G., Mazzanti A., McCanta A.C., Agarwal P.P., Arscott P., Dellefave-Castillo L.M., Vorovich E.E. (2020). Desmoplakin Cardiomyopathy, a Fibrotic and Inflammatory Form of Cardiomyopathy Distinct from Typical Dilated or Arrhythmogenic Right Ventricular Cardiomyopathy. Circulation.

[63] Verstraelen T.E., Van Lint F.H., Bosman L.P., De Brouwer R., Proost V.M., Abeln B.G., Taha K., Zwinderman A.H., Dickhoff C., Oomen T. (2021). Prediction of ventricular arrhythmia in phospholamban p.Arg14del mutation carriers–reaching the frontiers of individual risk prediction. Eur. Heart J..

[64] Bermúdez-Jiménez F.J., Carriel V., Brodehl A., Alaminos M., Campos A., Schirmer I., Milting H., Abril B., Álvarez M., López-Fernández S. (2018). Novel Desmin Mutation p.Glu401Asp Impairs Filament Formation, Disrupts Cell Membrane Integrity, and Causes Severe Arrhythmogenic Left Ventricular Cardiomyopathy/Dysplasia. Circulation.

[65] Thiene G., Corrado D., Nava A., Rossi L., Poletti A., Boffa G.M., Daliento L., Pennelli N. (1991). Right ventricular cardiomyopathy: Is there evidence of an inflammatory aetiology?. Eur. Heart J..

[66] Groeneweg J.A., van der Zwaag P.A., Olde Nordkamp L.R., Bikker H., Jongbloed J.D., Jongbloed R., Wiesfeld A.C., Cox M.G., van der Heijden J.F., Atsma D.E. (2013). Arrhythmogenic Right Ventricular Dysplasia/Cardiomyopathy According to Revised 2010 Task Force Criteria with Inclusion of Non-Desmosomal Phospholamban Mutation Carriers. Am. J. Cardiol..

[67] Te Rijdt W.P., Ten Sande J.N., Gorter T.M., van der Zwaag P.A., van Rijsingen I.A., Boekholdt S.M., van Tintelen J.P., van Haelst P.L., Planken R.N., de Boer R.A. (2019). Myocardial fibrosis as an early feature in phospholamban p.Arg14del mutation carriers: Phenotypic insights from cardiovascular magnetic resonance imaging. Eur. Heart J. Cardiovasc. Imaging.

[68] Protonotarios A., Elliott P.M. (2019). Arrhythmogenic cardiomyopathies (ACs): Diagnosis, risk stratification and management. Heart.

[69] Taha K., E Verstraelen T., de Brouwer R., Bruin-Bon R.H.A.C.M.d., Cramer M.J., Rijdt W.P.T., Bouma B.J., A de Boer R., A Doevendans P., Asselbergs F.W. (2023). Optimal echocardiographic assessment of myocardial dysfunction for arrhythmic risk stratification in phospholamban mutation carriers. Eur. Heart J.-Cardiovasc. Imaging.

[70] Pinto Y.M., Elliott P.M., Arbustini E., Adler Y., Anastasakis A., Böhm M., Duboc D., Gimeno J., De Groote P., Imazio M. (2016). Proposal for a revised definition of dilated cardiomyopathy, hypokinetic non-dilated cardiomyopathy, and its implications for clinical practice: A position statement of the ESC working group on myocardial and pericardial diseases. Eur. Heart J..

[71] Merlo M., Cannatà A., Gobbo M., Stolfo D., Elliott P.M., Sinagra G. (2018). Evolving concepts in dilated cardiomyopathy. Eur. J. Heart Fail..

[72] Hulten E., Aslam S., Osborne M., Abbasi S., Bittencourt M.S., Blankstein R. (2016). Cardiac sarcoidosis—State of the art review. Cardiovasc. Diagn. Ther..

[73] Limongelli G., Adorisio R., Baggio C., Bauce B., Biagini E., Castelletti S., Favilli S., Imazio M., Lioncino M., Merlo M. (2022). Diagnosis and Management of Rare Cardiomyopathies in Adult and Paediatric Patients. A Position Paper of the Italian Society of Cardiology (SIC) and Italian Society of Paediatric Cardiology (SICP). Int. J. Cardiol..

[74] Vita T., Okada D.R., Veillet-Chowdhury M., Bravo P.E., Mullins E., Hulten E., Agrawal M., Madan R., Taqueti V.R., Steigner M. (2018). Complementary Value of Cardiac Magnetic Resonance Imaging and Positron Emission Tomography/Computed Tomography in the Assessment of Cardiac Sarcoidosis. Circ. Cardiovasc. Imaging.

[75] Ammirati E., Frigerio M., Adler E.D., Basso C., Birnie D.H., Brambatti M., Friedrich M.G., Klingel K., Lehtonen J., Moslehi J.J. (2020). Management of Acute Myocarditis and Chronic Inflammatory Cardiomyopathy. Circ. Heart Fail..

[76] Monda E., Limongelli G. (2022). Is There a Role for Genetic Testing in Patients With Myocarditis?. Circ. Genom. Precis. Med..

[77] van der Bijl P., Delgado V., Bootsma M., Bax J.J. (2018). Risk Stratification of Genetic, Dilated Cardiomyopathies Associated With Neuromuscular Disorders. Circulation.

[78] Kamdar F., Garry D.J. (2016). Dystrophin-Deficient Cardiomyopathy. J. Am. Coll. Cardiol..

[79] Yilmaz A., Gdynia H.-J., Baccouche H., Mahrholdt H., Meinhardt G., Basso C., Thiene G., Sperfeld A.-D., Ludolph A.C., Sechtem U. (2008). Cardiac involvement in patients with Becker muscular dystrophy: New diagnostic and pathophysiological insights by a CMR approach. J. Cardiovasc. Magn. Reson..

[80] Duan D., Goemans N., Takeda S., Mercuri E., Aartsma-Rus A. (2021). Duchenne muscular dystrophy. Nat. Rev. Dis. Primers.

[81] Palladino A., Papa A.A., Morra S., Russo V., Ergoli M., Rago A., Orsini C., Nigro G., Politano L. (2019). Are there real benefits to implanting cardiac devices in patients with end-stage dilated dystrophinopathic cardiomyopathy? Review of literature and personal results. Acta Myol..

[82] Blaszczyk E., Gröschel J., Schulz-Menger J. (2021). Role of CMR Imaging in Diagnostics and Evaluation of Cardiac Involvement in Muscle Dystrophies. Curr. Heart Fail. Rep..

[83] Nigro G., Comi L., Politano L., Bain R. (1990). The incidence and evolution of cardiomyopathy in Duchenne muscular dystrophy. Int. J. Cardiol..

[84] Luetkens J.A., von Landenberg C., Isaak A., Faron A., Kuetting D., Gliem C., Dabir D., Kornblum C., Thomas D. (2019). Comprehensive Cardiac Magnetic Resonance for Assessment of Cardiac Involvement in Myotonic Muscular Dystrophy Type 1 and 2 without Known Cardiovascular Disease. Circ. Cardiovasc. Imaging.

[85] Angulski A.B.B., Hosny N., Cohen H., Martin A.A., Hahn D., Bauer J., Metzger J.M. (2023). Duchenne muscular dystrophy: Disease mechanism and therapeutic strategies. Front. Physiol..

[86] Kerstens T.P., van Everdingen W.M., Habets J., van Dijk A.P., Helbing W.A., Thijssen D.H., Cate F.E.U.T. (2023). Left ventricular deformation and myocardial fibrosis in pediatric patients with Duchenne muscular dystrophy. Int. J. Cardiol..

[87] Marchel M., Madej-Pilarczyk A., Tymińska A., Steckiewicz R., Ostrowska E., Wysińska J., Russo V., Grabowski M., Opolski G. (2021). Cardiac Arrhythmias in Muscular Dystrophies Associated with Emerinopathy and Laminopathy: A Cohort Study. J. Clin. Med..

[88] Sanna T. (2003). Cardiac features of Emery–Dreifuss muscular dystrophy caused by lamin A/C gene mutations. Eur. Heart J..

[89] Wang S., Peng D. (2019). Cardiac Involvement in Emery-Dreifuss Muscular Dystrophy and Related Management Strategies. Int. Heart J..

[90] Heller S.A., Shih R., Kalra R., Kang P.B. (2020). Emery-Dreifuss muscular dystrophy. Muscle Nerve.

[91] Sanchez F., Weitz C., Gutierrez J.M., Mestroni L., Hanneman K., Vargas D. (2022). Cardiac MR Imaging of Muscular Dystrophies. Curr. Probl. Diagn. Radiol..

[92] Iavarone M., Covino S., Petillo R., Russo V. (2023). Interatrial block as a first clinical presentation of atrial cardiomyopathy related to a novel LMNA variant: A case report. Eur. Heart J.-Case Rep..

[93] Thornton C.A. (2014). Myotonic dystrophy. Neurol. Clin..

[94] Russo V., Rago A., Ciardiello C., Russo M.G., Calabrò P., Politano L., Nigro G. (2016). The Role of the Atrial Electromechanical Delay in Predicting Atrial Fibrillation in Myotonic Dystrophy Type 1 Patients. J. Cardiovasc. Electrophysiol..

[95] Russo V., Rago A., Papa A.A., Politano L., Golino P., Russo M.G., Calabro R., Nigro G. (2013). Does a high percentage of right ventricular pacing influence the incidence of paroxysmal atrial fibrillation in myotonic dystrophy type 1 patients?. Pol. Heart J..

[96] Russo V., Nigro G., Di Meo F., Papa A.A., Della Cioppa N., Proietti R., Russo M.G., Calabrò R., Politano L. (2014). The effect of atrial preference pacing on atrial fibrillation electrophysiological substrate in Myotonic Dystrophy type 1 population. Acta Myol..

[97] Russo V., Di Meo F., Rago A., A Papa A., Molino A., Mosella M., Politano L., Russo M.G., Nigro G. (2015). Paroxysmal atrial fibrillation in myotonic dystrophy type 1 patients: P wave duration and dispersion analysis. Eur. Rev. Med. Pharmacol. Sci..

[98] Russo V., Antonini G., Massa R., Casali C., Mauriello A., Martino A.M., Marconi R., Garibaldi M., Franciosa P., Zecchin M. (2024). Comprehensive Cardiovascular Management of Myotonic Dystrophy Type 1 Patients: A Report from the Italian Neuro-Cardiology Network. J. Cardiovasc. Dev. Dis..

[99] Russo V., Papa A.A., Nigro G. (2017). The Controversial Epidemiology of Left Ventricular Dysfunction in Patients with Myotonic Dystrophy Type 1. JAMA Cardiol..

[100] Groh W.J., Bhakta D., Tomaselli G.F., Aleong R.G., Teixeira R.A., Amato A., Asirvatham S.J., Cha Y.-M., Corrado D., Duboc D. (2022). 2022 HRS expert consensus statement on evaluation and management of arrhythmic risk in neuromuscular disorders. Heart Rhythm..

[101] Russo V., Sperlongano S., Gallinoro E., Rago A., Papa A.A., Golino P., Politano L., Nazarian S., Nigro G. (2020). Prevalence of Left Ventricular Systolic Dysfunction in Myotonic Dystrophy Type 1: A Systematic Review. J. Card. Fail..

[102] Russo V., Papa A.A., Rago A., Nigro G. (2016). Which Is the True Epidemiology of Atrial Fibrillation in Myotonic Dystrophy Type 1 Patients?. Pacing Clin. Electrophysiol..

[103] Petri H., Vissing J., Witting N., Bundgaard H., Køber L. (2012). Cardiac manifestations of myotonic dystrophy type 1. Int. J. Cardiol..

[104] Russo V., Capolongo A., Bottino R., Carbone A., Palladino A., Liccardo B., Nigro G., Marchel M., Golino P., D’andrea A. (2023). Echocardiographic Features of Cardiac Involvement in Myotonic Dystrophy 1: Prevalence and Prognostic Value. J. Clin. Med..

[105] Matsuda N.M., Oliveira R.B., Dantas R.O., Iazigi N. (1995). Effect of isosorbide dinitrate on gastroesophageal reflux in healthy volunteers and patients with Chagas’ disease. Dig. Dis. Sci..

[106] Marin-Neto J.A., Cunha-Neto E., Maciel B.C., Simões M.V. (2007). Pathogenesis of Chronic Chagas Heart Disease. Circulation.

[107] Rassi A., Rassi A., Marin-Neto J.A. (2009). Chagas heart disease: Pathophysiologic mechanisms, prognostic factors and risk stratification. Mem. Inst. Oswaldo Cruz.

[108] Rochitte C.E., Oliveira P.F., Andrade J.M., Ianni B.M., Parga J.R., Ávila L.F., Kalil-Filho R., Mady C., Meneghetti J.C., Lima J.A. (2005). Myocardial Delayed Enhancement by Magnetic Resonance Imaging in Patients with Chagas’ Disease. J. Am. Coll. Cardiol..

[109] Miranda C.H., Figueiredo A.B., Maciel B.C., Marin-Neto J.A., Simões M.V. (2011). Sustained Ventricular Tachycardia Is Associated with Regional Myocardial Sympathetic Denervation Assessed with ^123^I-Metaiodobenzylguanidine in Chronic Chagas Cardiomyopathy. J. Nucl. Med..

[110] Køber L., Thune J.J., Nielsen J.C., Haarbo J., Videbæk L., Korup E., Jensen G., Hildebrandt P., Steffensen F.H., Bruun N.E. (2016). Defibrillator Implantation in Patients with Nonischemic Systolic Heart Failure. N. Engl. J. Med..

[111] Beggs S.A.S., Jhund P.S., E Jackson C., McMurray J.J.V., Gardner R.S. (2018). Non-ischaemic cardiomyopathy, sudden death and implantable defibrillators: A review and meta-analysis. Heart.

[112] Corrado D., Leoni L., Link M.S., Della Bella P., Gaita F., Curnis A., Salerno J.U., Igidbashian D., Raviele A., Disertori M. (2003). Implantable Cardioverter-Defibrillator Therapy for Prevention of Sudden Death in Patients with Arrhythmogenic Right Ventricular Cardiomyopathy/Dysplasia. Circulation.

[113] Hulot J.S., Jouven X., Empana J.P., Frank R., Fontaine G. (2004). Natural History and Risk Stratification of Arrhythmogenic Right Ventricular Dysplasia/Cardiomyopathy. Circulation.

[114] Kumar S., Baldinger S.H., Gandjbakhch E., Maury P., Sellal J.-M., Androulakis A.F., Waintraub X., Charron P., Rollin A., Richard P. (2016). Long-Term Arrhythmic and Nonarrhythmic Outcomes of Lamin A/C Mutation Carriers. J. Am. Coll. Cardiol..

[115] Gigli M., Stolfo D., Graw S.L., Merlo M., Gregorio C., Nee Chen S., Dal Ferro M., PaldinoMD A., De Angelis G., Brun F. (2021). Phenotypic Expression, Natural History, and Risk Stratification of Cardiomyopathy Caused by Filamin C Truncating Variants. Circulation.

[116] Akhtar M.M., Lorenzini M., Pavlou M., Ochoa J.P., O’mahony C., Restrepo-Cordoba M.A., Segura-Rodriguez D., Bermúdez-Jiménez F., Molina P., Cuenca S. (2021). Association of Left Ventricular Systolic Dysfunction among Carriers of Truncating Variants in Filamin C With Frequent Ventricular Arrhythmia and End-stage Heart Failure. JAMA Cardiol..

[117] Hodgkinson K., Connors S., Merner N., Haywood A., Young T., McKenna W., Gallagher B., Curtis F., Bassett A., Parfrey P. (2013). The natural history of a genetic subtype of arrhythmogenic right ventricular cardiomyopathy caused by a p.S358L mutation in TMEM43. Clin. Genet..

[118] Hey T.M., Rasmussen T.B., Madsen T., Aagaard M.M., Harbo M., Mølgaard H., Møller J.E., Eiskjær H., Mogensen J. (2019). Pathogenic RBM20-Variants Are Associated with a Severe Disease Expression in Male Patients with Dilated Cardiomyopathy. Circ. Heart Fail..

[119] Wahbi K., BEN Yaou R., Gandjbakhch E., Anselme F., Gossios T., Lakdawala N.K., Stalens C., Sacher F., Babuty D., Trochu J.-N. (2019). Development and Validation of a New Risk Prediction Score for Life-Threatening Ventricular Tachyarrhythmias in Laminopathies. Circulation.

[120] Hodgkinson K.A., Howes A., Boland P., Shen X.S., Stuckless S., Young T.-L., Curtis F., Collier A., Parfrey P.S., Connors S.P. (2016). Long-Term Clinical Outcome of Arrhythmogenic Right Ventricular Cardiomyopathy in Individuals with a p.S358L Mutation in *TMEM43* Following Implantable Cardioverter Defibrillator Therapy. Circ. Arrhythm. Electrophysiol..

[121] Gigli M., Merlo M., Graw S.L., Barbati G., Rowland T.J., Slavov D.B., Stolfo D., Haywood M.E., Ferro M.D., Altinier A. (2019). Genetic Risk of Arrhythmic Phenotypes in Patients with Dilated Cardiomyopathy. J. Am. Coll. Cardiol..

[122] A McDonagh T., Metra M., Adamo M., Gardner R.S., Baumbach A., Böhm M., Burri H., Butler J., Čelutkienė J., Chioncel O. (2021). Corrigendum to: 2021 ESC Guidelines for the diagnosis and treatment of acute and chronic heart failure: Developed by the Task Force for the diagnosis and treatment of acute and chronic heart failure of the European Society of Cardiology (ESC) With the special contribution of the Heart Failure Association (HFA) of the ESC. Eur. Heart J..

[123] Gilljam T., Haugaa K.H., Jensen H.K., Svensson A., Bundgaard H., Hansen J., Dellgren G., Gustafsson F., Eiskjær H., Andreassen A.K. (2018). Heart transplantation in arrhythmogenic right ventricular cardiomyopathy—Experience from the Nordic ARVC Registry. Int. J. Cardiol..

[124] Russo V., Ciabatti M., Brunacci M., Dendramis G., Santobuono V., Tola G., Picciolo G., Teresa L.M., D’andrea A., Nesti M. (2023). Opportunities and drawbacks of the subcutaneous defibrillator across different clinical settings. Expert Rev. Cardiovasc. Ther..

[125] AlTurki A., Proietti R., Russo V., Dhanjal T., Banerjee P., Essebag V. (2019). Anti-arrhythmic drug therapy in implantable cardioverter-defibrillator recipients. Pharmacol. Res..

[126] Oloriz T., Silberbauer J., Maccabelli G., Mizuno H., Baratto F., Kirubakaran S., Vergara P., Bisceglia C., Santagostino G., Marzi A. (2014). Catheter Ablation of Ventricular Arrhythmia in Nonischemic Cardiomyopathy. Circ. Arrhythm. Electrophysiol..

[127] Proietti R., Russo V., AlTurki A. (2019). Anti-arrhythmic therapy in patients with non-ischemic cardiomyopathy. Pharmacol. Res..

[128] Sethi Y., Patel N., Kaka N., Kaiwan O., Kar J., Moinuddin A., Goel A., Chopra H., Cavalu S. (2023). Precision Medicine and the future of Cardiovascular Diseases: A Clinically Oriented Comprehensive Review. J. Clin. Med..

[129] Leopold J.A., Loscalzo J. (2018). Emerging Role of Precision Medicine in Cardiovascular Disease. Circ. Res..

[130] Musunuru K. (2017). Genome Editing: The Recent History and Perspective in Cardiovascular Diseases. J. Am. Coll. Cardiol..

[131] Sethi Y., Mahtani A.U., Khehra N., Padda I., Patel N., Sebastian S.A., Malhi G., Kaiwan O., Saith S., Johal G. (2023). Gene Editing as the Future of Cardiac Amyloidosis Therapeutics. Curr. Probl. Cardiol..

[132] Ma H., Marti-Gutierrez N., Park S.-W., Wu J., Lee Y., Suzuki K., Koski A., Ji D., Hayama T., Ahmed R. (2017). Correction of a pathogenic gene mutation in human embryos. Nature.

